# Mood Disorder in Cancer Patients Undergoing Radiotherapy During the COVID-19 Outbreak

**DOI:** 10.3389/fpsyg.2021.568839

**Published:** 2021-03-19

**Authors:** Valerio Nardone, Alfonso Reginelli, Claudia Vinciguerra, Pierpaolo Correale, Maria Grazia Calvanese, Sara Falivene, Angelo Sangiovanni, Roberta Grassi, Angela Di Biase, Maria Angela Polifrone, Michele Caraglia, Salvatore Cappabianca, Cesare Guida

**Affiliations:** ^1^Unit of Radiation Oncology, Ospedale del Mare, Naples, Italy; ^2^Department of Precision Medicine, University of Campania “Luigi Vanvitelli,” Naples, Italy; ^3^Unit of Neurology, University Hospital of Salerno, Salerno, Italy; ^4^Unit of Medical Oncology, Grand Metropolitan Hospital “Bianchi Melacrino Morelli,” Reggio Calabria, Italy

**Keywords:** mood disorders, COVID-19, radiotherapy, cancer, anxiety, depression

## Abstract

**Introduction:** Novel coronavirus (COVID-19) is having a devastating psychological impact on patients, especially patients with cancer. This work aims to evaluate mood disorders of cancer patients undergoing radiation therapy during COVID-19 in comparison with cancer patients who underwent radiation therapy in 2019.

**Materials and Methods:** We included all the patients undergoing radiation therapy at our department in two-time points (once a week for a month in May 2019) and during the COVID-19 outbreak (in April 2020). All the patients were asked to fulfill a validated questionnaire (STAI-Y1, State trait anxiety inventory scale), the Symptom Distress thermometer (SDT) (from 0 to 10 score), and the Beck Depression Inventory v.2 (BDI-2). We took into account the COVID-19 outbreak and also sex, age, week of radiation treatment, and disease.

**Results:** We included 458 patients (220 males and 238 females), with a median age of 64 years. STAI-Y1 median score was 40 (mean 41,3, range 19–79), whereas the median score of SDT was five and BDI-2 median score was 11. STAI-Y1, SDT, and BDI-2 were significantly correlated with the COVID-19 outbreak (*p* < 0,001 for all the tests), sex (*p*: 0,016 for STAI-Y1, *p* < 0.001 for SDT, *p*:0.013 for BDI-2), week of treatment (*p*: 0.012 for STAI-Y1 and *p*: 0.031 for SDT), and disease (*p*:0.015 for STAI-Y1, *p* < 0.001 for SDT and *p*:0.020 for BDI-2).

**Conclusions:** The prevalence of mood disorders in patients undergoing radiation therapy is higher than expected and even higher during the COVID-19 outbreak. These measurements could be useful as a baseline to start medical humanities programs to decrease these scores.

## Introduction

Since WHO announced the novel coronavirus (COVID-19) outbreak as a pandemic on 11th March, the virus has reached more than 4 million cases and 300,000 deaths all over the world (Laghi and Grassi, 2020; Neri et al., 2020).

Notably, the psychological effects of COVID-19 on both patients and healthcare workers could be serious and deserves a systematic investigation (Belfiore et al., 2020; Huang and Zhao, 2020; Tsamakis et al., 2020).

A cancer diagnosis often implies extensive emotional, physical, and social suffering, therefore current cancer management should incorporate different psychosocial interventions to improve patients' quality of life (Zimmermann-Schlegel et al., 2017; Senf et al., 2019).

In this context, it is easy to imagine the potential threats of the COVID-19 outbreak on the psychological well-being of cancer patients.

Currently the radiotherapy community is focused on providing responses to face the different issues of this critical period (Coles et al., 2020; Grassi et al., 2020; Guckenberger et al., 2020; Rinaldi et al., 2020; Scorsetti et al., 2020; Zaorsky et al., 2020), but the management of psychological disorders has not been evaluated yet.

This work aims at the prevalence of mood disorders (anxiety, distress, and depression) for cancer patients undergoing radiation therapy during the COVID-19 outbreak in comparison with patients treated in 2019.

## Materials and Methods

### Population

The Institutional Review Board (IRB) approved the survey and protocol.

Patients undergoing RT were prospectively enrolled in the present study in two-time points (once a week for a month in May 2019) and during the COVID-19 outbreak (once, in April 2020).

Inclusion criteria were as follows: written informed consent concerning treatment risk, psychological test agreement, and age >18 years.

### Procedure

In the two time periods chosen, all the patients that underwent radiotherapy on the days when the tests were collected (once a week for a month for time point 1 and once for period 2) were included in the present evaluation. During treatment all the patients are visited every week from the clinician, in order to evaluate acute toxicity and radiotherapy side effects. Before the visit, the patients performed a self-administered psychological evaluation according to the described instruments. Demographic and clinical variables registered were sex, age, disease, and week of radiation treatment.

### Instruments

Three validated patient-reporting tests were used: the State version of anxiety inventory scale (STAI-Y1), the Symptom Distress Thermometer (SDT), and the Beck Depression Inventory vers.2 (BDI-2).

The STAI-Y1 is a self-report measure with 20 items to assess state anxiety, with each item evaluated on a 4-point Likert scale. A cutoff point of 40 has been suggested to detect clinically significant symptoms for scale (Knight et al., 1983; Julian, 2011).

The SDT is an 11-point scale with endpoints labeled “no distress” (0 points) and “extreme distress” (10 points) (Distress Management, 2003).

For SDT, a cutoff point of 4 was chosen, based on the scores of the Hospital Anxiety and Depression Scale and Brief Symptom Inventory 18 (Jacobsen et al., 2005).

The BDI-2 is a validated tool for patient mood assessment and has been developed to investigate the presence and degree of depressive symptoms.

The BDI-2 is a 21-item self-administered questionnaire, with each response scored on a scale of 1–3. All the scores are summed to give the BDI score. A BDI-2 score of 0–13 indicates minimal symptoms, 14–19 mild symptoms, 20–28 moderate symptoms, and 29–63 severe symptoms (Beck et al., 1996).

### Statistical Methods

Differences in patients' characteristics (sex, age, disease, week of treatment) between the two time points were evaluated with the Chi-square test in order to compare the two cohorts of patients (see Table 1).

**Table 1:** Characteristics of the two cohorts of timepoints.

**Parameters**	**Time point 1 (Pre Covid-19)**	**Time point 2 (Covid-19 Outbreak)**	**Statistical analysis (Chi-square test)**
**Sex**			
Males	181 (47.6%)	39 (50%)	*p*:0.711
Females	199 (52.4%)	39 (50%)	
**Age**			
<50 years	50 (13.2%)	13 (16.7%)	*p* < 0.001
50–70 years	192 (50.5%)	58 (74.4%)	
>70 years	138 (36.3%)	7 (9%)	
**Disease**			
Gastrointestinal	51 (13.4%)	6 (7.7%)	*p*:0.543
Brain	39 (10.3%)	6 (7.7%)	
Breast	121 (31.8%)	30 (38.5%)	
Lung	15 (3.9%)	6 (7.7%)	
Prostate	58 (15.3%)	13 (16.7%)	
Head and Neck	66 (17.4%)	12 (15.4%)	
Other/Palliative	30 (7.9%)	5 (6.4%)	
**Week of treatment**			
First week	87 (22.9%)	20 (25.6%)	*p*:0.659
Other	293 (77.1%)	58 (74.4%)	

#### Univariate Analysis

The three scales were analyzed as continuous data. Student's *t*-test was used in univariate analysis to assess differences in scales according to patient-specific variables (sex, age, week of treatment) and between the two timepoints (pre and during COVID-19).

A sensitivity analysis was performed considering the three scales as categorical items with cut-offs corresponding to those indicated for each instrument. Chi square test was used to assess association with the two timepoints.

#### Multivariate Analysis

All the parameters (sex, age, week of treatment, COVID timepoint) were considered in a linear regression analysis with a stepwise method. For the linear regression analysis, the nominal parameter “disease” was categorized as a dummy variable.

The three scales were considered dependent variables and the parameters sex, age, week of treatment, disease, and timepoints were considered as independent variables.

A two-tailed *p*-value < 0.05 was considered statistically significant.

All the statistical analysis was performed on SPSS v.23.0.

## Results

### Population

We included 458 patients (220 males and 238 females), with a median age of 64 years (mean 63,9 years, range 29–88 years), tested before COVID-19 (380 patients, 83%) and during the COVID-19 outbreak (78 patients, 17%) (see Table 1 for the characteristics of enrolled patients).

The two cohorts of patients showed a significant difference in terms of age, probably due to the selection of patients during the COVID-19 outbreak.

### Instruments

STAI-Y1 mean score was 41.3 (standard deviation 10.89), with 227 patients (49.6%) showing a STAI-Y1 <40.

SDT mean score was 4.6 (standard deviation 2.55), with 211 patients (46.1%) showing a low anxiety score on SDT (<4).

BDI-2 mean score was 13.8 (standard deviation 10.10). A total of 297 patients were categorized as minimal score (64.8%), 80 as mild score (17.5%), 46 as moderate score (10%), and 35 as severe score (7.6%).

Differences were found in all three scores between the two timepoints, highlighting the worsening of mood disorders during the COVID-19 pandemic (see Table 2).

**Table 2:** Differences among the three tests in the two cohorts of timepoints.

**Parameters**	**Time point 1 (Pre Covid-19)**	**Time point 2 (Covid-19 Outbreak)**	*P*-value^*^
**STAI-Y1**			
**(Continuous data)**			
Mean	40.24	46.51	*p* < 0.001
Standard Deviation	10.39	11.82	
**SDT**			
**(Continuous data)**			
Mean	4.35	5.88	*p* < 0.001
Standard Deviation	2.50	2.42	
**BDI-2**			
**(Continuous data)**			
Mean	13.27	16.7	*p*:0.005
Standard Deviation	10.02	10.06	
**STAI-Y1**			
**(Categorical)**			
<40	216 (56.8%)	29 (37.2%)	*p*: 0.0015
≥40	164 (43.2%)	49 (62.8%)	
**SDT**			
**(Categorical)**			
<4	184 (48.4%)	27 (34.6%)	*p*: 0.025
≥4	196 (51.6%)	51 (65.4%)	
**BDI-2**			
**(Categorical)**			
Minimal score	262 (68.9%)	35 (44.9%)	*P* < 0.001
Mild score	57 (15%)	23 (29.5%)	
Moderate score	33 (8.7%)	13 (16.7%)	
Severe score	28 (7.4%)	7 (9%)	

* *P-value was relative to Student's t-test for continuous variables and chi-square test for categorical items*.

### Univariate Analysis

A linear regression model was used to evaluate the association between each instrument and the other variables considered.

STAI-Y1 was correlated with the COVID-19 outbreak (*p* < 0.001) being higher post COVID-19 outbreak, sex (*p*:0.016) being higher for female patients, and week of treatment (*p*:0.012) being higher in the first week of treatment.

SDT, similarly, was correlated with the COVID-19 outbreak (*p* < 0.001), sex (*p* < 0.001), and week of treatment (*p*:0.036).

BDI-2, finally, was correlated with the COVID-19 outbreak (*p*:0.005) and sex (*p*: 0.013).

### Multivariate Analysis

Multivariable linear regression analysis showed that all the significant parameters associated with STAI-Y1 also maintained their significance when considered in the same model: COVID-19 outbreak (*p* < 0.001), week of treatment (*p*: 0.001), and sex (*p*: 0.015).

**Figure 1:**
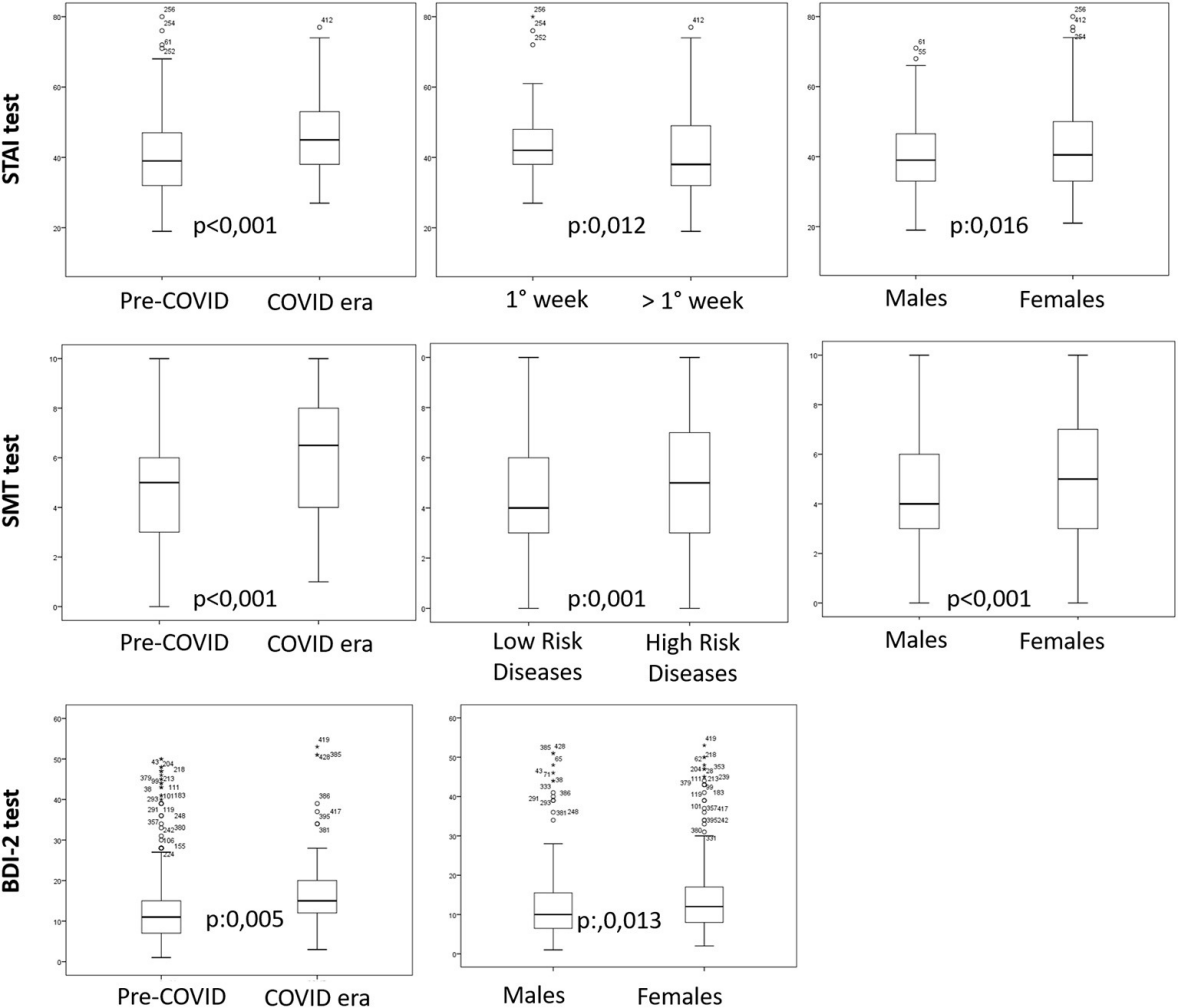
Boxplots of the parameters that garnered a significant result in the multivariate analysis for the three tests (STAI-Y1 test, SMT test, and BDI-test). High-risk diseases included brain cancer, head and neck cancer, and lung cancer. Low-risk diseases included all the remaining cancer diseases.

For SDT, similarly, the only significant parameters are COVID-19 (*p* < 0.001), disease (*p*:0.001), and sex (*p*:0.004). The diseases that showed a higher SDT were brain cancer, head and neck cancer, and lung cancer, whereas all the remaining cancer diseases showed a lower SDT (see Figure 1).

For BDI-2 the only significant parameters remain COVID-19 outbreak (*p*: 0.004) and sex (*p*:0.011) (see Table 3; Figure 1).

**Table 3:** Linear regression analysis was used to correlate all the variables with the test STAI-Y1, SDT, and BDI-2.

**Dependent (Linear regression)**	**Parameter**	***p*-value**	**B**
**STAI-Y1**	(constant)		
	**Covid-19**	** < 0.001**	0.24
	**Week of treatment**	**0.001**	−0.15
	**Sex**	**0.015**	0.10
	Age	0.112	0.07
	Disease	0.070	−0.08
**SDT**	(constant)		
	**Covid-19**	** <0.001**	0.26
	**Disease**	**0.001**	−0.15
	**Sex**	**0.004**	0.13
	Age	0.803	0.01
	Week of treatment	0.057	−0.11
**BDI-2**	(constant)		
	**Covid-19**	**p:0.004**	0.13
	**Sex**	**p:0.011**	0.12
	Age	0.826	−0.01
	Week of treatment	0.951	−0.01
	Disease	0.565	−0.03

*Bold values are significant parameters*.

## Discussion

The psychological distress related to the diagnosis of cancer can be devastating for the patients and their relatives (Lim et al., 2013), with responses that include denial of the diagnosis, fear of death, fear of recurrence of cancer, concerns about body image, as well as impacts on sexuality, relationships, and lifestyle (Zabora et al., 2001; Schouten et al., 2019).

A mood disorder may be part of the reaction to the news of a cancer diagnosis, but in many patients it will persist, causing an additional burden of disease (Hopwood and Stephens, 2000).

Conversely, the illness itself or the cancer treatments may lead to a radical modification of patients' everyday life activities, especially in cases of advanced illness (Zaza et al., 2005). These conditions can have a relevant impact on patients' quality of life (Tang et al., 2017) and eventually induce mood disorders such as psychological distress, anxiety, and depression (Andersen et al., 1984; Andersen and Tewfik, 1985; Stiegelis et al., 2004; Bradt and Dileo, 2010).

Cancer patients undergoing radiotherapy represent an even more fragile population that is associated with increased levels of anxiety and depression that is often under-detected and undertreated (Stiegelis et al., 2004), as frequently this population of patients has not regained the optimal psychological and physical conditions from previous treatments (de Graeff et al., 2000; Monga et al., 2005). A significant percentage of radiotherapy patients, in fact, is subjected to different types of therapies in the previous months (such as surgery possibly followed by chemotherapy in a subset of breast cancer patients, induction chemotherapy in a subset of lung or head and neck cancer, and so on).

At the same time, early alarming reports have suggested that patients with cancer seem more likely to develop severe COVID-19 (Liang et al., 2020), and patients undergoing radiotherapy are also required to make daily visit hospitals for some weeks, with an increased risk of contagion.

Salari et al. have recently performed a meta-analysis to investigate the prevalence among the general population of stress, anxiety, and depression (Salari et al., 2020). The authors found that the prevalence of these disorders was, respectively, 29.6, 31.9, and 33.7%, so it is essential to develop psychological interventions to improve the mental health of the population during the pandemic. Xiong et al., in a similar study, found that the risk factors associated with the mood disorders include female gender, younger age (<40 years), presence of chronic illnesses, student status, and frequent exposure to press news concerning COVID-19 (Xiong et al., 2020).

Vindegaard et al., conversely, investigated the consequences of COVID-19 on mental health and found lower psychological well-being and higher scores of anxiety and depression vs. before the pandemic, with no differences among the initial phases of the outbreak to a month later (Vindegaard and Benros, 2020). Poor self-related health was, again, recognized as a risk factor with higher risk of mood disorders.

The points just discussed can shed light on our results which show that during the critical period of the COVID-19 outbreak patients undergoing RT develop increased depression, anxiety, and distress, according to all the tests used.

The incidence of depression in cancer patients varies considerably among the different studies, ranging from 7 to 49% (Derogatis et al., 1983; Jenkins et al., 1998; Kai-hoi Sze et al., 2000; Pascoe et al., 2000).

Hahn et al. performed a routine screening for depression in radiation oncology patients and they found that only 15% of patients endorsed significant depressive symptoms (Hahn et al., 2004). Conversely, Kawase et al. investigated a homogeneous cohort of 172 patients with early-stage breast cancer and found that 42% of the patients showed depressive disorder (Kawase et al., 2012).

Alacacioglu et al. also investigated depression and anxiety levels in cancer patients and discovered that nearly half of the patients showed mild and severe depression (respectively 29.1 and 18.2%) (Alacacioglu et al., 2013). Both depression and anxiety were higher in women, in people with low socioeconomic level, and in patients with a relapsing disease.

Katz et al., conversely, investigated the depression in head and neck cancer patients undergoing radiotherapy and found that the prevalence of Major and Minor Depression was 20% (Katz et al., 2004).

The RTOG 0841 trial has recently investigated the use of screening for depression in cancer patients receiving radiotherapy in a multi-institutional setting (Wagner et al., 2017). The cohort of patients included 455 patients with different diseases; 75 patients (16.5%) exceeded screening cut-offs for depressive symptoms and were further investigated.

Our results, thus, are consistent with the literature, although the prevalence of depression among cancer patients is variable among the different studies, due to the choice of tests adopted and the differences in the cohorts of analyzed patients.

In regard to anxiety, a review of RT studies indicated that a significant percentage of patients showed clinically significant levels of anxiety at the initiation of RT (Stiegelis et al., 2004; León-Pizarro et al., 2007; Halkett et al., 2016).

Literature has also demonstrated that anxiety due to RT is ranked first among the factors influencing patients' adherence to treatment (Dragomir and Fodoreanu, 2013; Ho et al., 2013; Hyphantis et al., 2013).

Voigtmann et al. have investigated anxiety in a cohort of 240 patients undergoing RT, and found that 28% of the patients scored in the pathological or borderline anxiety range (Voigtmann et al., 2010).

Nixon et al. have recently investigated the anxiety due to the immobilization mask used for RT in head and neck cancer patients and found that females were more likely to experience higher mask anxiety (Nixon et al., 2019). The population of the patients analyzed in our study show a big proportion of patients with brain cancer and head and neck cancer (123 pts, 26.8%), thus explaining the high anxiety levels in the study.

Marital status, conversely, is not correlated with the development of anxiety (Nieder and Kämpe, 2018), whereas Shimotsu et al. have classified specific types of anxiety, respectively due to adverse effects of RT, the environment of RT, and treatment effects of RT (Shimotsu et al., 2010).

In this regard, Mullaney et al. have correlated the department's psychosocial climate and treatment environment on patients' anxiety during radiotherapy, and found that both these aspects significantly impact anxiety levels (Mullaney et al., 2016).

The other parameters that are correlated with mood disorders are sex, week of treatment, and disease.

Female patients show more anxiety than male patients, in accordance with previous studies (Dunn et al., 2012). Increased anxiety levels in some specific diseases (head and neck cancer, brain cancer, and lung cancer) may be due to the more severe conditions and to the use of immobilization systems such as masks, as previously reported.

Finally, patients show increased anxiety at the beginning of the RT, whereas the anxiety levels tend to decrease in the following week. This trend is in line with the literature (Dunn et al., 2012).

### Limitations

This study must recognize several limitations. First of all, the study utilized a small group from a single institution. In addition, worries about identification and potential medical insights may have induced participants to score low on tests.

All cancer patients were tested before the COVID-19 outbreak to obtain basal values and to explore different strategies with the aim to improve radiation therapy workplace cultures, such as medical humanities programs and music therapy (Vinciguerra et al., 2019; Nardone et al., 2020).

The basal test has allowed a first and unprecedented measurement of the real effects of COVID-19 outbreak in cancer patients.

Finally, it is important to underline that this study was conducted after the peak period of the COVID-19 outbreak, at the end of April. Future research is required in different institutions at different time points. At the same time, it is pivotal to follow up with the analyzed population.

## Conclusions

Cancer patients have shown a significant increase in anxiety and depression due to the COVID-19 outbreak.

Multi-institutional prospective evaluation is needed to confirm these data and to develop proper strategies in order to mitigate the increase.

## Data Availability Statement

The raw data supporting the conclusions of this article will be made available by the authors, without undue reservation.

## Ethics Statement

The studies involving human participants were reviewed and approved by the Institutional Ethics Committee of Asl Napoli 1 (Naples, Italy). The patients/participants provided their written informed consent to participate in this study.

## Author Contributions

VN, CV, MGC, SF, and RG contributed to conception and design of the study. VN, PC, AD, and MP organized the database and performed the statistical analysis. VN, MC, SC, and CG wrote the first draft of the manuscript. CV, MGC, SF, and RG wrote sections of the manuscript. All authors contributed to manuscript revision, read, and approved the submitted version.

## Conflict of Interest

The authors declare that the research was conducted in the absence of any commercial or financial relationships that could be construed as a potential conflict of interest.
